# miR-200c-3p Regulates Epitelial-to-Mesenchymal Transition in Epicardial Mesothelial Cells by Targeting Epicardial Follistatin-Related Protein 1

**DOI:** 10.3390/ijms22094971

**Published:** 2021-05-07

**Authors:** Elena Pontemezzo, Eleonora Foglio, Enza Vernucci, Alessandra Magenta, Marco D’Agostino, Sara Sileno, Elena Astanina, Federico Bussolino, Laura Pellegrini, Antonia Germani, Matteo Antonio Russo, Federica Limana

**Affiliations:** 1 Department of Experimental Medicine, Sapienza University of Rome, 00161 Rome, Italy; pontemezzo90@gmail.com (E.P.); eleonora.foglio83@gmail.com (E.F.); enza.vernucci@gmail.com (E.V.); laura_pellegrini@hotmail.it (L.P.); 2 Experimental Immunology Laboratory, Istituto Dermopatico dell’Immacolata, IDI-IRCCS, Via dei Monti di Creta 104, 00167 Rome, Italy; ale.magenta@gmail.com (A.M.); marcodagostino86@hotmail.it (M.D.); sara.sileno@libero.it (S.S.); antoniagermani@yahoo.com (A.G.); 3 Institute of Translational Pharmacology (IFT), Consiglio Nazionale delle Ricerche (CNR), Via Fosso del Cavaliere 100, 00133 Rome, Italy; 4 Department of Oncology, University of Turin, 10060 Candiolo, Italy; elena.astanina@ircc.it (E.A.); federico.bussolino@ircc.it (F.B.); 5 Candiolo Cancer Institute-FPO-IRCCS, 10060 Candiolo, Italy; 6 IRCCS San Raffaele Pisana and MEBIC Consortium, 00166 Rome, Italy; matteo.russo@sanraffaele.it; 7 Department of Human Science and Promotion of the Quality of Life, San Raffaele Roma Open University, 00166 Rome, Italy; 8 Laboratory of Cellular and Molecular Pathology, IRCCS San Raffaele Pisana, 00166 Rome, Italy

**Keywords:** epicardial EMT, EMCs, miR200c, FSTL1, molecular rehabilitation

## Abstract

Recent findings suggest that epithelial to mesenchymal transition (EMT), a key step during heart development, is involved in cardiac tissue repair following myocardial infarction (MI). MicroRNAs (miRNAs) act as key regulators in EMT processes; however, the mechanisms by which miRNAs target epicardial EMT remain largely unknown. Here, by using an *in vitro* model of epicardial EMT, we investigated the role of miRNAs as regulators of this process and their potential targets. EMT was induced in murine epicardial-mesothelial cells (EMCs) through TGF β1 treatment for 48, 72, and 96 h as indicated by the expression of EMT-related genes by qRT-PCR, WB, and immunofluorescence. Further, enhanced expression of stemness genes was also detected. Among several EMT-related miRNAs, miR-200c-3p expression resulted as the most strongly suppressed. Interestingly, we also found a significant upregulation of Follistatin-related protein 1 (FSTL1), a miR-200c predicted target already identified as a potent cardiogenic factor produced by epicardial cells that promotes regeneration following MI. Dual-luciferase reporter assay demonstrated that miR-200c-3p directly targeted the 3′-untranslated region of FSTL1 in EMCs. Consistently, WB analysis showed that knockdown of miR-200c-3p significantly increased FSTL1 expression, whereas overexpression of miR-200c-3p counteracted TGF β1-mediated FSTL1 upregulation. Importantly, FSTL1 silencing maintained epithelial features in EMCs, despite EMT induction by TGF β1, and attenuated EMT-associated traits, including migration and stemness. In conclusion, epicardial FSTL1, an important cardiogenic factor in its secreted form, induces EMT, stemness, and migration of EMCs in a miR-200c-3p dependent pathway.

## 1. Introduction

Emerging evidence suggests that epithelial to mesenchymal transition (EMT) might contribute directly to stem cell phenotypes both in cancer stem cells and in stem cells from adult tissues, including the heart [1,2,3]. In the adult human heart, c-kit^+^ cells have been detected in the subepicardium of patients with ischemic cardiomyopathy [4]. These cells derive from epicardial EMT as shown by the simultaneous expression of epithelial and mesenchymal markers, with an increase in mesenchymal markers in hearts with ischemic cardiomyopathy compared to normal hearts [5].

In the adult murine heart, following infarction, epicardial cells undergo EMT, giving rise to c-kit^+^ cells whose differentiation into the main cardiac cell lineages is induced by activation of fetal epicardial genes [6]. These results were supported by another more recent study demonstrating that exosomal clusterin, isolated from the pericardial fluid of patients with acute MI, improved cardiac function in mice following MI in part by promoting the formation of c-kit^+^ cells through epicardial EMT [3].

Therefore, understanding the mechanisms that induce epicardial EMT following MI might help to find new targeted treatment strategies to support cardiac repair.

microRNAs (miRNAs) are small non-coding RNAs that post-transcriptionally regulate gene expression [7], and they are involved in the regulation of both cardiac development and injury [8,9]. Furthermore, miRNAs are known to act as key regulators in EMT processes [10]. Considering epicardial EMT, it has been demonstrated that miR-31 inhibited fibrogenic EMT in TGF β1-stimulated epicardial mesothelial cells (EMCs) by targeting Islet-1, while miR-21 promoted it by involving Programmed Cell Death 4 and Sprouty Homolog 1 [11,12]. In vivo, let-7 inhibition in the infarcted mouse heart resulted in functional benefits due to an increase in epicardial EMT. These results were supported by gain-and loss-of-function studies in epicardial cells *in vitro* [13].

miR-200c has been implicated as a major modulator of EMT in cancer cell lines [14,15], but its involvement in cardiac EMT remains to be determined. In the heart, miR-200c has been involved in ischemia and reperfusion injury by targeting the glutaminase-mediated glutamine metabolism [16]. Further, its inhibition protects cardiomyocytes from hypoxia-induced apoptosis via GATA4 [17] and attenuates cardiomyocyte hypertrophy in high glucose-treated cardiomyocytes [18].

Follistatin-like 1 (FSTL1) is a secreted glycoprotein, member of the SPARC (Secreted Protein Acid Rich in Cysteine) family of proteins abundantly expressed in multiple embryonic and adult tissues and with multiple effects [19]. Previous studies have identified FSTL1 as a protective cardiokine during post-MI cardiac remodeling [20,21,22] and pathological cardiac hypertrophy [23]. Specifically, it has been shown that increasing levels of FSTL1 in the heart resulted in a cardioprotective effect against ischemic injury due to enhanced cardiomyocyte survival and stimulated angiogenesis [20,22]. Further, a recent study demonstrated that FSTL1 is also an intrinsic cardiokine promoting survival and proliferation of hypoxic MSCs, thereby prolonging their survival and retention after transplantation in the infarcted murine heart [24]. Nevertheless, Wei and colleagues showed that the epicardial origin of FSTL1 appears to be key for inducing its regenerative rather than antiapoptotic response [21]. Accordingly, their study demonstrated that epicardial FSTL1 expression disappeared from the infarcted area in response to ischemic damage and its reconstitution by a bioengineered collagen patch loaded with purified human FSTL1 and sutured to the epicardial surface of the infarcted heart of mice, resulted in improved survival and cardiac function by a reduction in scarring, an increase in the formation of new vessels, and an increase in the number of dividing cardiomyocytes.

The aim of the present study was to determine the possible role of miRNAs in an *in vitro* model of epicardial EMT. Our results showed that TGF β1-induced EMT of epicardial mesothelial cells (EMCs) is significantly modulated by miR-200c through targeting FSTL1. Therefore, miR-200c represents a promising target to manipulate epicardial cell fate and, potentially, cardiac repair and regeneration.

## 2. Results

### 2.1. TGF β1 Treatment Induces Mesenchyme Formation and Stem Cell-Like Acquisition in EMCs

Recently, it has been demonstrated that in vitro cultures of EMCs undergo EMT in response to different inducers, with the potency being TGF β1 > TNF-α >> IL-1β [12].

**Figure 1:**
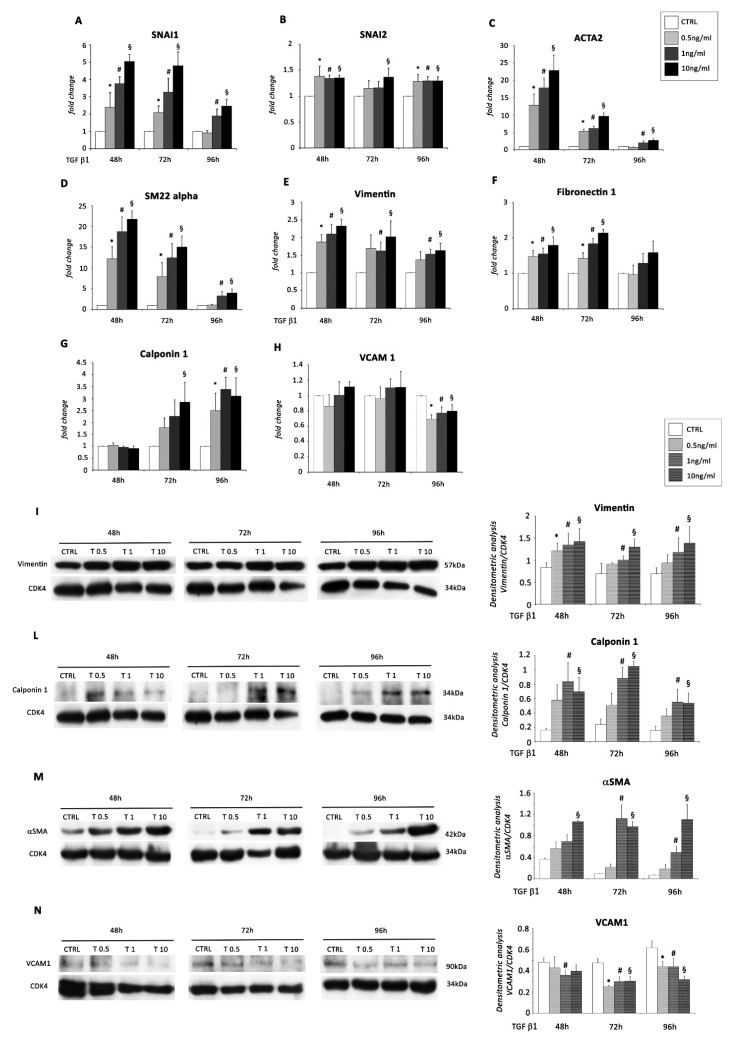
TGF β1 treatment induces EMT in EMCs. EMCs were incubated with 0.5, 1, and 10 ng/mL of TGF β1 for 48, 72, and 96 h. mRNA expression levels for (**A**) SNAI1, (**B**) SNAI2, (**C**) ACTA2, (**D**) SM22alpha, (**E**) Vimentin, (**F**) Fibronectin 1, (**G**) Calponin 1 and (**H**) VCAM1 were determined by real time PCR. The bar graphs show fold increases in expression of the studied genes in EMCs with respect to control untreated cells, set at 1. Data were normalized to GAPDH, a housekeeping gene, and represent means ± SEM of three separate experiments, each repeated in triplicate. *, *p* < 0.05 for 0.5 ng/mL of TGF β1; #, *p* < 0.05 for 1 ng/mL of TGF β1; §, *p* < 0.05 for 10 ng/mL of TGF β1 vs. control conditions (CTRL). Western blot analysis showing the expression of (**I**) Vimentin, (**L**) Calponin 1, (**M**) αSMA, and (**N**) VCAM1, in EMCs stimulated with 0.5, 1, and 10 ng/mL of TGF β1 for 48, 72, and 96 h compared to control conditions (CTRL). The same filter was probed with anti-CDK4 pAb to show the equal loading. Left panel: A representative Western blotting of three independent experiments is shown. Right panel: Densitometric analysis of Western blot. Data are shown as means ± SEM. *, *p* < 0.05 for 0.5 ng/mL of TGF β1; #, *p* < 0.05 for 1 ng/mL of TGF β1; §, *p* < 0.05 for 10 ng/mL of TGF β1 vs. control conditions (CTRL).

Accordingly, we incubated EMCs (described in Figure S1) with TGF β1, at three different concentrations, for 48, 72, and 96 h. EMT was observed (Figure S2A), and EMCs showed robust activation of Smad2 and Smad3 by phosphorylation following stimulation with all doses of TGF β1 at all time points (Figure S2B). Importantly, TGF β1 stimulation resulted in the modulation of several EMT transcripts. Specifically, the pattern of expression of SNAI1, SNAI2 (markers of EMT during embryogenesis), ACTA2, SM22 alpha, Vimentin, Calponin 1, and Fibronectin 1 (mesenchymal markers) were determined by qRT-PCR at all three time points. The analysis showed a significant up-regulation of SNAI1 at all time points by stimulation with 1 and 10 ng/mL of TGF β1 (Figure 1A); about SNAI2, we detected a significant up-regulation at 48 and 96 h of treatment with all doses of TGF β1, while at 72 h, only the highest dose was able to induce a significant increase in the mRNA levels (Figure 1B). The expression of mesenchymal markers increased, showing a maximal induction at all doses of TGF β1 after 48 h of treatment (Figure 1C–F) except for Calponin 1, whose expression was highest and significant only at the latest time point (Figure 1G). The expression of the epithelial marker VCAM-1 was significantly reduced by all doses of TGF β1 only after 96 h of treatment (Figure 1H).

For several EMT markers, increased protein expression levels were obtained by WB analysis after TGF β1 treatment mainly at 72 and 96 h at the two highest concentrations (Figure 1 I–M). In addition, protein expression of VCAM1 significantly decreased at all TGF β1 doses following 72 and 96 h of treatment (Figure 1N).

**Figure 2:**
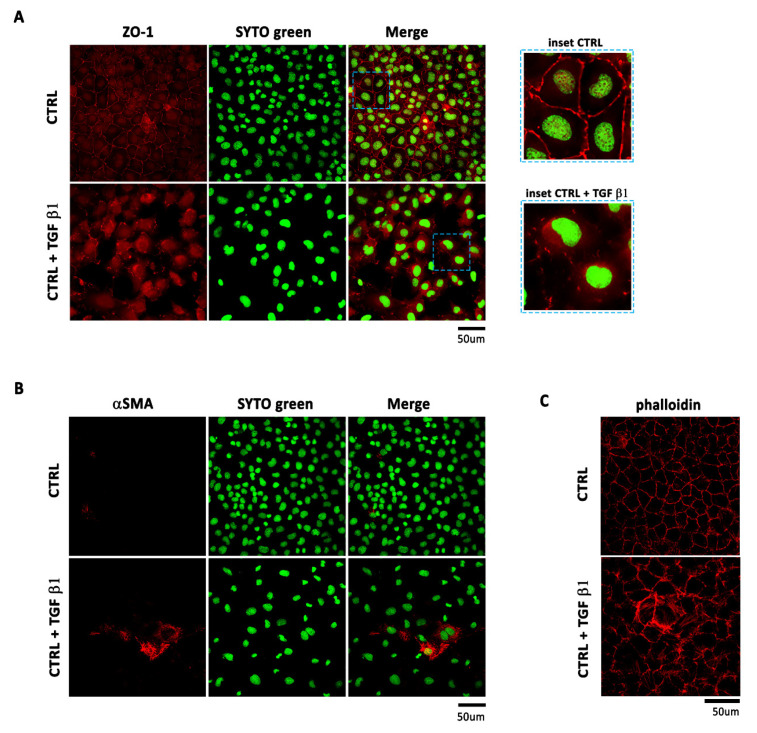
*TGF β1 treatment induces loss of epithelial character and acquisition of mesenchymal character in EMCs*. Non-confluent EMC cultures were stimulated with TGF β1 (1 ng/mL) and examined after 48 h by immunofluorescence for (**A**) the epithelial marker ZO-1 (inset: magnified version) and (**B**) the mesenchymal marker αSMA. Left panel: red fluorescence indicates ZO-1 or αSMA. Central panel: green fluorescence indicates SYTO Green Fluorescent staining of nuclei. Right panel: merge of both images. Scale Bar: 50 µm. After 48 h stimulation with TGF β1, (**C**) phalloidin visualized the increase in actin filaments.

To further confirm the occurrence of EMT, we stained EMCs with the epicardial and epithelial adhesion molecule ZO-1 and with the mesenchymal marker αSMA. In untreated cells, ZO-1 was localized at the cell junction (Figure 2A) while αSMA was not detected (Figure 2B). When stimulated with TGF β1, EMCs were elongate and had lost cell–cell contacts and ZO-1 expression at the cell–cell junction (Figure 2A) while they showed increased staining of αSMA, therefore confirming the mesenchymal transition (Figure 2B). In particular, upon TGF β1 stimulation, EMCs showed a reorganization of actin into stress fibers necessary to allow actin-myosin based contraction (Figure 2C).

**Figure 3:**
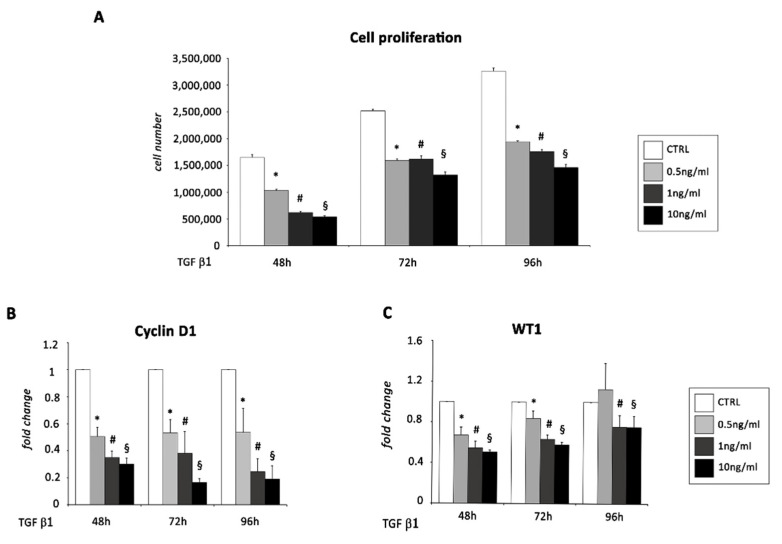
*EMCs undergo EMT after exposure to TGF β1*. Growth curve of EMCs after (**A**) 48 h, 72, and 96 h of culture and 0.5, 1, and 10 ng/mL of TGF β1 stimulation, compared to untreated conditions. Data represent means ± SEM of three Scheme 0. for 0.5 ng/mL of TGF β1; #, *p* < 0.05 for 1 ng/mL of TGF β1; §, *p* < 0.05 for 10 ng/mL of TGF β1 vs. control conditions (CTRL). mRNA expression levels of (**B**) Cyclin D1 and (**C**) WT1 in EMCs following exposure to 0.5, 1, and 10 ng/mL of TGF β1 for 48, 72, and 96 h, determined by real time PCR. The bar graphs show fold increases in expression of the studied genes in EMCs with respect to control untreated cells, set at 1. Data were normalized to GAPDH, a housekeeping gene, and represent means ± SEM of three separate experiments, each repeated in triplicate. *, *p* < 0.05 for 0.5 ng/mL of TGF β1; #, *p* < 0.05 for 1 ng/mL of TGF β1; §, *p* < 0.05 for 10 ng/mL of TGF β1 vs. control conditions (CTRL).

In accordance with EMT progression, we detected a significant reduction in cell number after 48, 72, and 96 h of TGF β1 incubation (Figure 3A) and a parallel decrease in mRNA expression of Cyclin D1, an important regulator of cell cycle progression, in treated EMCs up to 96 h of TGF β1 treatment (Figure 3B).

Indeed, over time, treated EMCs were also characterized by a loss of the epicardial marker WT1 (Figure 3C) that, in primary epicardial cells, has been demonstrated to be necessary for the maintenance of the epicardial phenotype, whereas its knockdown enhanced the mesenchymal morphology [25].

**Figure 4:**
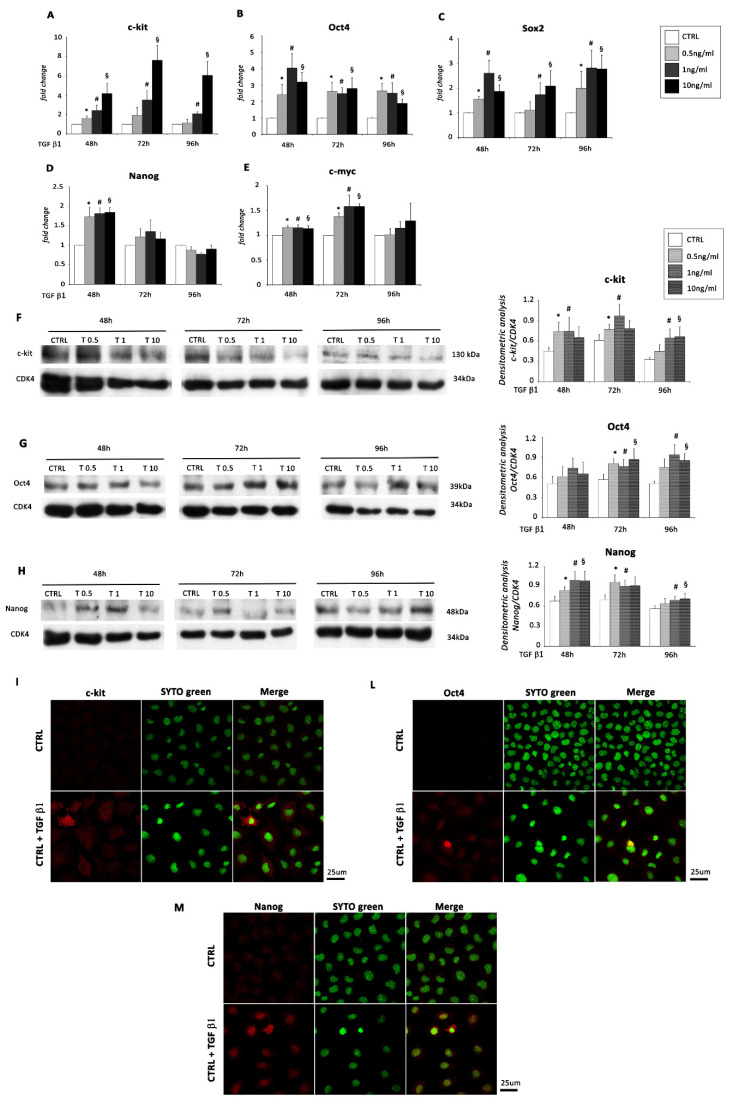
EMCs express stem cell-like markers after exposure to TGF β1. EMCs were incubated with 0.5, 1, and 10 ng/mL of TGF β1 for 48, 72, and 96 h. mRNA expression levels for (**A**) c-kit, (**B**) Oct4, (**C**) Sox2, (**D**) Nanog, and (**E**) c-myc were determined by real time PCR. The bar graphs show fold decreases or increases in expression of the studied genes in EMCs with respect to control untreated cells, set at 1. Data were normalized to GAPDH, a housekeeping gene, and represent means ± SEM of three separate experiments, each repeated in triplicate. *, *p* < 0.05 for 0.5 ng/mL of TGF β1; #, *p* < 0.05 for 1 ng/mL of TGF β1; §, *p* < 0.05 for 10 ng/mL of TGF β1 vs. control conditions (CTRL). Western blot analysis showing the expression of (**F**) c-kit, (**G**) Oct4, and (**H**) Nanog, in EMCs stimulated with 0.5, 1, and 10 ng/mL of TGF β1 for 48, 72, and 96 h compared to control conditions (CTRL). The same filter was probed with anti-CDK4 pAb to show the equal loading. Left panel: A representative Western blotting of three independent experiments is shown. Right panel: Densitometric analysis of Western blot. Data are shown as means ± SEM. *, *p* < 0.05 for 0.5 ng/mL of TGF β1; #, *p* < 0.05 for 1 ng/mL of TGF β1; §, *p* < 0.05 for 10 ng/mL of TGF β1 vs. control conditions (CTRL). Non-confluent EMC cultures were stimulated with TGF β1 (1 ng/mL) and examined after 48 h by immunofluorescence for the stem cell markers (**I**) c-kit, (**L**) Oct4, and (**M**) Nanog. Left panel: red fluorescence indicates c-kit, Oct4, or Nanog. Central panel: green fluorescence indicates SYTO Green Fluorescent staining of nuclei. Right panel: merge of both images. Scale Bar: 25 µm.

It has been demonstrated that EMT of cancer cells determines the formation of cancer stem cells with enhanced migratory capacity and invasiveness [26]. Epicardial EMT drives to the formation of progenitors [27]. Our group recently provided evidence of epicardial EMT-mediated formation of c-kit^+^ progenitor cells *in vivo* [3]. Accordingly, TGF β1 treatment of EMCs increased the expression of stem cell genes. Specifically, mRNA expression for c-kit, Oct4, and Sox2 significantly increased at all three time points (Figure 4A–C) and mainly at all doses, while Nanog expression strongly increased only at the earliest time point (Figure 4D) and c-myc expression levels significantly increased at 48 and 72 h (Figure 4E). Increased protein expression levels were obtained by WB analysis after TGF β1 stimulation at 48, 72, and 96 h for c-kit and Nanog (Figure 4F,H) and only at 72 and 96 h for Oct4 (Figure 4G). These results were also supported by immunofluorescence stainings (Figure 4I–M).

### 2.2. miR-200c Is the Major Modulated miRNA during TGF β1-Mediated EMT of EMCs

Results obtained indicated that our model could be used for further screening of EMT-related miRNAs.

**Figure 5:**
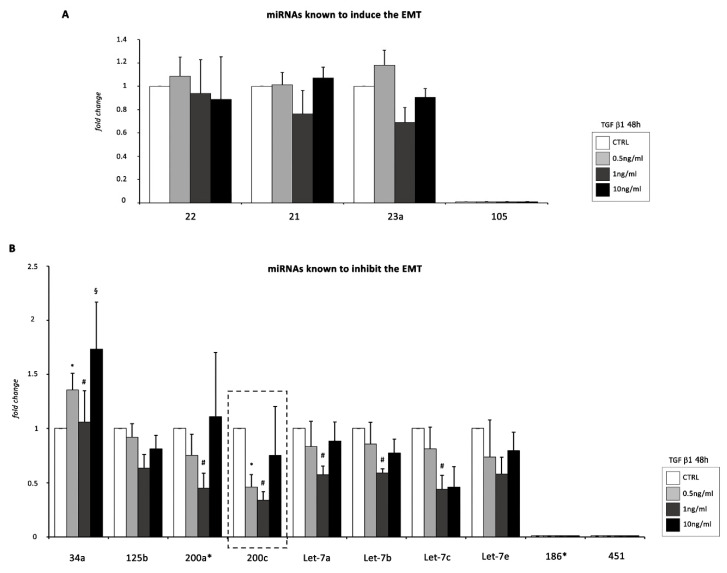
Screening of different miRNA implicated in TGF β1-induced EMT of EMCs. (**A**) Panel of miRNAs that induces the EMT. (**B**) Panel of miRNAs that inhibits the EMT. The level of expresScheme 202. (endogenous control) and represent means ± SEM of three separate experiments, each repeated in triplicate. *, *p* < 0.05 for 0. 5 ng/mL of TGF β1; #, *p* < 0.05 for 1 ng/mL of TGF β1; §, *p* < 0.05 for 10 ng/mL of TGF β1 vs. control conditions (CTRL).

As an initial step toward identifying miRNAs that may be implicated in TGF β1-induced EMT of EMCs, we focused on the expression of several miRNAs mainly involved in epicardial EMT [11,12,13], but also in the EMT of other cell types [10] (Figure 5A,B).

In our EMT model, qRT-PCR results indicated that five miRNAs (miR-200a, miR-200c, let-7a, let-7b, and let-7c), whose inhibition promotes EMT, were significantly downregulated in EMCs following 48 h of TGF β1 treatment (Figure 5B).

Interestingly, among these five miRNAs, miR-200c was by far the most down-modulated miRNA following incubation with 0.5 and 1 ng/mL of TGF β1 for 48 h. Therefore, we selected miR-200c for further investigation.

It is noteworthy that miR-34a, mostly involved in inhibiting the progression of tumor-associated EMT, was strongly up-regulated in our EMT model (Figure 5B).

By qRT-PCR, we verified the up-regulation of miR-200c main targets, i.e., ZEB1 and ZEB2 (Figure S3A,B) after 48, 72, and 96 h of TGF β1 treatment and at all doses: the up-regulation of ZEB1 was significant, mainly after 48 h of TGF β1 stimulation, while the modulation of ZEB2 showed a trend that was never significant. qRT-PCR results were confirmed by WB analysis, showing a strong enhancement in ZEB1 protein expression, but at a later time point (72 h instead of 48 h) (Figure S3C).

### 2.3. FSTL1 Is Induced in TGF β1-Mediated EMT of EMCs

Among miR-200c predicted targets, we found FSTL1. Since it has been recently documented the importance of epicardial FSTL1 in cardiac regeneration [21] and its involvement in airway and cancer EMT [28,29], we hypothesized that (1) FSTL1 could be also involved in epicardial EMT (2) through a miR-200c-dependent pathway.

**Figure 6:**
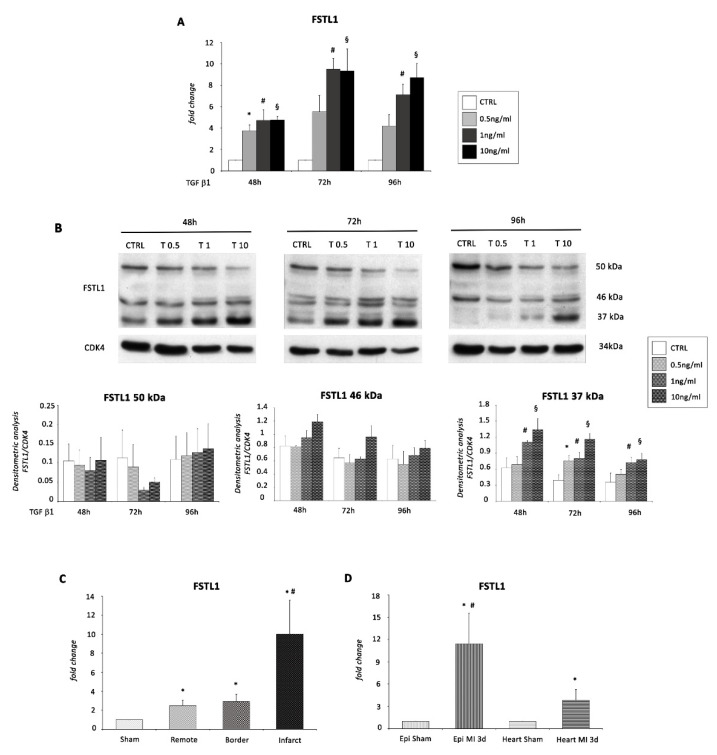
FSTL1 is a potential target of miR-200c. (**A**) mRNA expression and (**B**) protein expression of FSTL1 were determined in EMCs exposed to 48, 72, and 96 h of treatment with TGF β1 at three different concentrations (0.5, 1, and 10 ng/mL) compared to control conditions (CTRL). (**B**) WB analysis revealed the presence of three bands of approximately 37, 46, and 50 kDa representing the non-glycosylated, lower and higher glycosylated states of the detected FSTL1, respectively. *, *p* < 0.05 for 0.5 ng/mL of TGF β1; #, *p* < 0.05 for 1 ng/mL of TGF β1; §, *p* < 0.05 for 10 ng/mL of TGF β1 vs. control conditions (CTRL). (**C**) FSTL1 mRNA determined by real time PCR in the LV of sham operated mice, in the remote zone, in the border zone and in the infarcted region of three-day infarcted hearts (*n* = 3; *, *p* < 0.05 vs. sham; #, *p* < 0.05 vs. border). (**D**) FSTL1 mRNA expression determined by real time PCR in epicardial cells and in the myocardial tissue deprived of the epicardium from sham-operated hearts (epi Sham and heart Sham) and infarcted hearts (epi MI 3d and heart MI 3d), 3 days following MI. Data were normalized to GADPH and represent means ± SEM (*n* = 3, each *n* = pool of 6 animals *, *p* < 0.05 vs. sham. #, *p* < 0.05 vs. heart MI 3d).

We first investigated whether the expression levels of FSTL1 mRNA and protein were modulated in EMC cells following TGF β1 treatment. FSTL1 mRNA level was increased by 4-fold at 48 h and approximately 8-fold at 72 and 96 h upon treatment of EMCs with 1 and 10 ng/mL of TGF β1 (Figure 6A) confirming that FSTL1 is induced by TGF β1 signaling [30] also in our *in vitro* model. Interestingly, WB analysis revealed the presence of three bands for FSTL1: a protein band of 37 kDa corresponding to the non-glycosylated FSTL1 (FSTL1 37 kDa), a protein band of 50 kDa corresponding to the hyperglycosylated FSTL1 (FSTL1 50 kDa), and a protein band of 46 kDa corresponding to a smaller FSTL1 with, most likely, a single glycosylation site (FSTL1 46 kDa) (Figure 6B). In our experimental model, the non-glycosylated FSTL1 was the only one to be significantly modulated at all three time points. Specifically, the protein expression level of FSTL1 37 kDa was strongly up-regulated upon TGF β1 treatment, mainly at 48 h followed by 72 and 96 h (Figure 6B).

To test our hypothesis *in vivo*, we used a model of murine acute MI and first collected the remote zone, the region bordering the infarction, and the infarcted region from three day-infarcted hearts and the left ventricle (LV) from sham-operated mice. The pattern of expression of FSTL1 was determined by qRT-PCR. The analysis revealed that FSTL1 mRNA was detectable in sham operated control hearts and was induced 3 days following MI in all the three zones examined, but with the higher induction in the infarcted region (Figure 6C).

Then, to verify whether this up-regulation was specifically present in the epicardium, we isolated epicardial cells and the tissue of the LV deprived of the epicardium both from infarcted hearts, 3 days after coronary ligation, and from sham-operated controls. Interestingly, the level of FSTL1 mRNA expression was nearly 4-fold higher in the infarcted heart (deprived of the epicardium) compared to the control, but resulted 11-fold higher in epicardial cells from infarcted hearts compared to epicardial cells from sham controls (Figure 6D).

### 2.4. miR-200c Inhibits EMT by Targeting FSTL1

Once addressed the induction of FSTL1 in TGF β1-mediated EMT of EMCs, we investigated whether FSTL1 could be a direct target of miR-200c. Several studies already demonstrated the presence of multiple miRNA-binding sites in the 3′UTR of FSTL1 mRNA and their role in the inhibition of FSTL1 expression [31,32,33,34].

**Figure 7:**
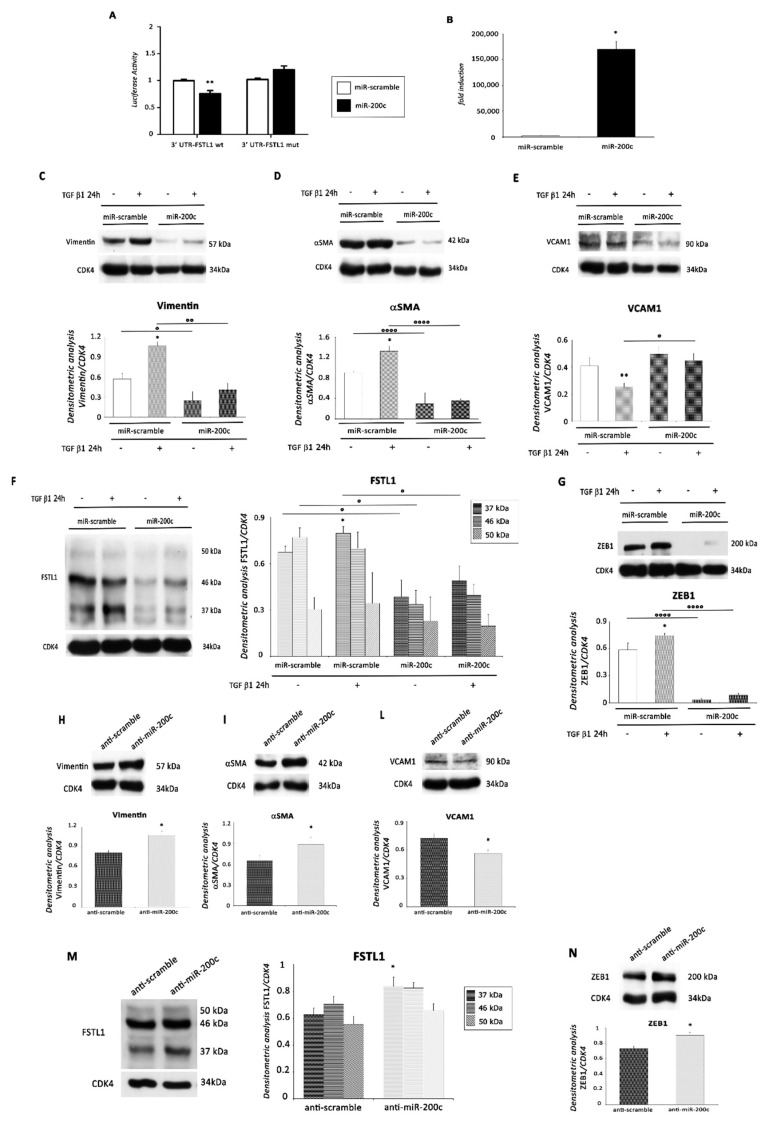
miR-200c inhibits the EMT by Targeting FSTL1. (**A**) FSLT1 is a direct target of miR-200c. HEK 293 were transfected with firefly luciferase constructs containing the murine 3′UTR- FSTL1 wt or the 3′UTR sequence mutated in miR-200c seed sequence (3′UTR-FSTL1 mut) both cloned in pEZX-MT06 plasmid. These constructs were co-transfected with a plasmid encoding either miR-200c or a miR-scramble sequence as a control. Values were normalized according to renilla luciferase activity (*n* = 5 in triplicate; **, *p* <0.001). miR-200c down-modulated the luciferase activity of the 3′UTR wt construct but not that of the mutated construct. (**B**–**G**) EMC cells were infected with a lentivirus encoding miR-200c or with a control virus. After 24 h, cells were selected with puromycin and plated. (**B**) miR-200c expression levels in EMCs infected with control virus or with the lentivirus encoding miR-200c. *, *p* < 0.05 The cells were then treated with TGF β1 (1 ng/mL) for 24 h and subjected to WB analysis of the EMT specific markers (**C**) Vimentin, (**D**) αSMA, and (**E**) VCAM1 and the direct targets (**F**) FSTL1 and (**G**) ZEB1. Upper panel (or left panel for FSTL1): A representative Western blotting of three independent experiments is shown. Lower panel (or right panel for FSTL1): Densitometric analysis of Western blot. Data are shown as means ± SEM. *, *p* < 0.05 vs. miR-scramble, two-tailed Student’s *t*-test; °, *p* < 0.05, °°, *p* < 0.005, °°°°, *p* < 0.0001, two-way ANOVA, Bonferroni post hoc test. (**H**–**N**) EMC cells were also infected with a lentivirus encoding either anti-miR-200c sequence or a scramble sequence. The cells were subjected to WB analysis of the EMT specific markers (**H**) Vimentin, (**I**) αSMA, and (**L**) VCAM1, and the direct targets (**M**) FSTL1 and (**N**) ZEB1. Upper panel (or left panel for FSTL1): A representative Western blotting of three independent experiments is shown. Lower panel (or right panel for FSTL1): Densitometric analysis of Western blot. Data are shown as means ± SEM. *, *p* < 0.05 vs. anti-scramble.

*In silico* analysis indicated that murine FSTL1 is a potential target of miR-200c. FSTL1 3′UTR is 2590 bp long and contains a miR-200c seed sequence in position 93–99 nt of the 3′UTR. In order to analyze this potential miRNA binding site, we assayed the luciferase activity of the construct containing the sequence 3′UTR of FSTL1 (3′UTR-FLST1 wt) and the same seed sequence mutated in miR-200c (3′UTR-FLST1 mut). miR-200c was able to decrease the luciferase activity of 3′UTR-FLST1 wt compared to scramble control, but not the one of the 3′UTR-FSLT1 mut (Figure 7A).

Taken together, these results show that FSTL1 is a direct target of miR-200c.

To investigate whether miR-200c inhibits EMT by targeting FSTL1, EMCs were infected either with a lentivirus encoding miR-200c or with a control virus (Figure 7B). Following infection, EMCs were treated with TGF β1 for 24 h. Stimulation of EMT by TGF β1 treatment was abrogated in EMCs by overexpression of miR-200c as demonstrated by the modulation of three EMT markers (Figure 7C–E). Specifically, the protein expression levels of Vimentin and αSMA (Figure 7C,D) were significantly inhibited, while that one of VCAM1 was strongly enhanced (Figure 7E). Importantly, overexpression of miR-200c induced a significant down-regulation of FSTL1 protein level in lenti-miR-200c transfected cells compared to control cells both in the absence and in the presence of TGF β1 treatment (Figure 7F). Again, the modulation was significant for FSTL1 37 kDa, but not for the other forms (Figure 7F). As expected, we also observed a significant protein down-modulation of ZEB1 in miR-200c overexpressing cells following TGF β1 treatment compared to control cells (Figure 7G). 

We also infected EMCs with a lentivirus encoding either anti-miR-200c sequence or a scramble sequence. The protein expression level of Vimentin and αSMA was significantly enhanced in transfected cells compared to control cells (Figure 7H,I). Accordingly, we also found a significant decrease of VCAM1 protein expression (Figure 7L). Anti-miR-200c treatment was able to induce an increase of FSTL1 protein levels in EMCs. It is noteworthy that FSTL1 37 kDa was the only form to be significantly modulated (Figure 7M). Lastly, the expression of ZEB1 was higher in EMC cells after miR-200c knockdown compared to control cells, as demonstrated by WB analysis (Figure 7N).

### 2.5. FSTL1 Is Required and Sufficient for the Acquisition of EMT-Associated and Stem-Like Traits

**Figure 8:**
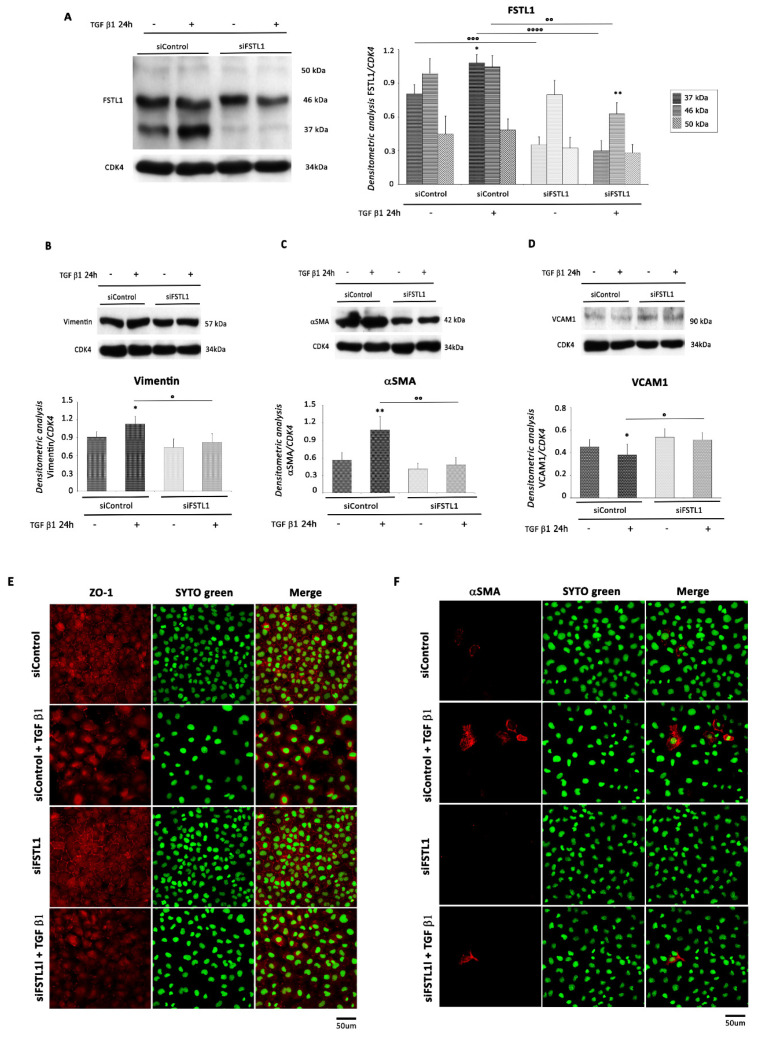
FSTL1 knockdown reverses EMT. EMC cells were transfected with control siRNA (siControl) or siRNA directed against FSTL1 (siFSTL1) and then treated or not with TGF β1 (1 ng/mL) for additional 24 h starting from the day after transfection. WB analysis performed with antibodies against (**A**) FSTL1, (**B**) Vimentin, (**C**) αSMA, and (**D**) VCAM1. CDK4 was used as loading control. Upper panel (or left panel for FSTL1): A representative Western blotting of three independent experiments is shown. Lower panel (or right panel for FSTL1): Densitometric analysis of Western blot. Data are shown as means ± SEM. *, *p* < 0.05 vs. siControl, **, *p* < 0.005 vs. siFSTL, two-tailed Student’s *t*-test; °, *p* < 0.05, °°, *p* < 0.005, °°°, *p* < 0.0007, °°°°, *p* < 0.0001, two-way ANOVA, Bonferroni post hoc test. (**E**,**F**) Representative images of EMCs immunostained for the epithelial marker ZO-1 and the mesenchymal marker αSMA, untreated and treated with TGF β1 (1 ng/mL) for 24 h and transfected with control siRNA (siControl) or siRNA directed against FSTL1 (siFSTL1). Left panel: red fluorescence indicates ZO-1 or αSMA. Central panel: green fluorescence indicates SYTO Green Fluorescent staining of nuclei. Right panel: merge of both images. Scale Bar: 50 µm.

To investigate whether FSTL1 regulates EMT and EMT-associated traits in EMCs, we transiently silenced its expression by siRNA (Figure 8A). We found that FSTL1 knockdown down-regulated the expression of mesenchymal markers and up-regulated VCAM1 expression (Figure 8B–D). These results were confirmed by immunofluorescence for ZO-1 and αSMA. Specifically, stimulation of EMT and formation of mesenchymal cells by treatment with TGF β1 was abrogated by FSTL1 knockdown (Figure 8E,F). 

**Figure 9:**
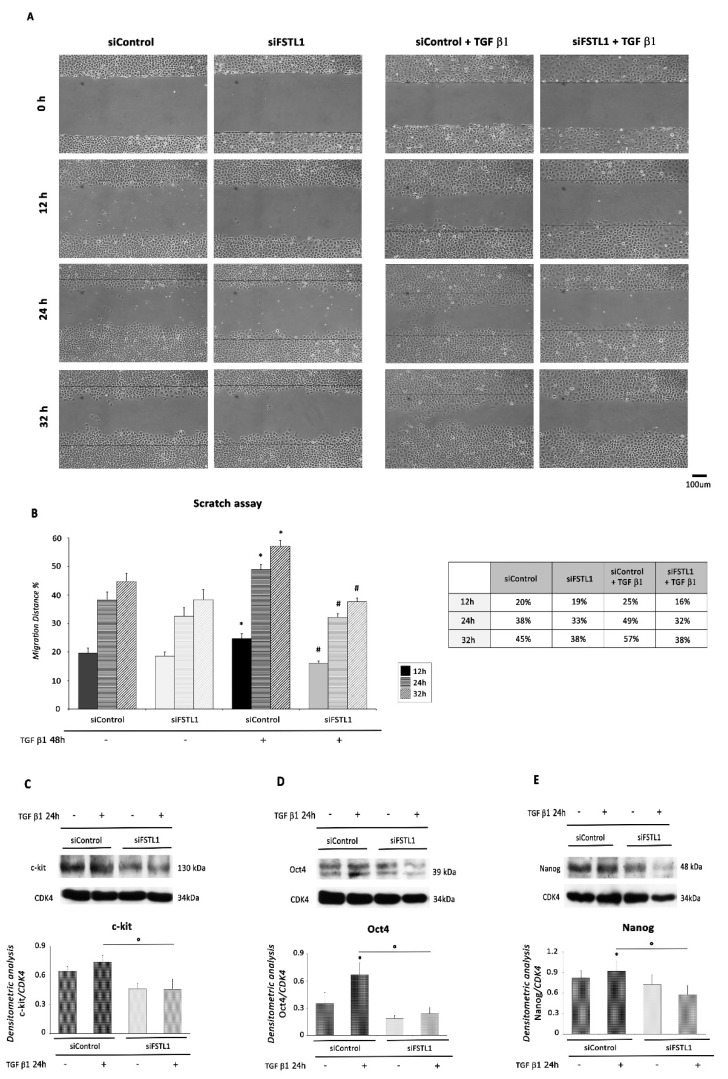
FSTL1 knockdown reverses EMT-associated and stem-like traits. (**A**) Representative images at 0, 12, 24, and 32 h after the scratch was made in EMCs transfected with siRNAs (siFSTL1 or siControl) for 72 h and treated or not with 1 ng/mL TGF β1 for additional 48 h starting from the day after transfection. Scale bar: 100 µm. (**B**) Quantification of migration distance, given as the percentage of wound healing calculated on the ratio between wound width at the observed time point and wound width at the 0 h time point (*n* = 3; *, *p* < 0.05 vs. siControl; #, *p* < 0.05 vs. siControl + TGF β1 48 h). (**C**–**E**) WB analysis performed with antibodies against (**C**) c-kit, (**D**) Oct4, and (**E**) Nanog. CDK4 was used as loading control. Upper panel: A representative Western blotting of three independent experiments is shown. Lower panel: Densitometric analysis of Western blot. Data are shown as means ± SEM. *, *p* < 0.05 vs. siControl, two-tailed Student’s *t*-test; °, *p* < 0.05, two-way ANOVA, Bonferroni post hoc test.

Importantly, since cell motility is known to increase following the mesenchymal transition, cell migratory activity was examined using the scratch assay. After 48 h, the scratch had completely closed (not shown), and the migration distance was significantly decreased at 12, 24, and 32 h in TGF β1 treated cells, but not in control or FSTL1-knockdown cells with or without TGF β1 treatment (Figure 9A,B), indicating that FSTL1 knockdown inhibited the migration of EMC cells.

Finally, FSTL1 knockdown by siRNA inhibited the expression of c-kit, Oct4, and Nanog in EMCs compared to control cells. Importantly, TGF β1-stimulated siFSTL1 EMC cells exhibited a significant decrease in protein expression levels of the same stem cell markers compared to TGF β1-treated control cells (Figure 9C–E).

## 3. Discussion

In the present study, we have identified FSTL1 as a potential EMT regulator of EMC cells lining the epicardium and miR-200c as an upstream miRNA that directly targets FSTL1.

We have recently demonstrated that exosomal clusterin, identified in the pericardial fluid of patients with acute MI, determined an improvement in cardiac function in part by EMT-mediated epicardial activation. Therefore, a deeper understanding of the molecular mechanisms that induce epicardial EMT and CPC generation would undoubtedly help to direct the balance in favor of the endogenous regenerative capacities of the adult heart rather than the formation of scar tissue. Since overwhelming evidence in the literature, as well as experimental studies, have shown miRNAs to regulate EMT, we investigated which miRNAs could be involved in this process using an *in vitro* model of epicardial EMT.

Specifically, TGF β1 treatment resulted in EMT induction and increased expression of stem cell markers in EMCs. When we tried to identify miRNAs that may be implicated in this process, surprisingly, we did detect only a slight induction (at the highest TGF β1 concentration) in the expression of miR-21, previously identified as a key regulator of fibrogenic EMT of EMCs [12] while miR-200c resulted strongly inhibited. It is noteworthy that the miR-200 family and in particular miR-200c has been highly studied in terms of development, stemness, proliferation, EMT, therapy resistance, and metastasis [14,35]. In particular, inhibition of its expression induces EMT and stemness in different types of cancer cells [15,16]. In the heart, miR-200c has been demonstrated to exacerbate the ischemia/reperfusion injury through targeting the glutaminase-mediated glutamine metabolism and its inhibition has been found to protect cardiomyocytes from hypoxia-induced apoptosis by targeting GATA-4 [17,28].

In our *in vitro* model, miR-200c manipulation showed substantial impact on EMC phenotype since its overexpression markedly inhibited the expression of EMT and stem cell markers while its silencing with an anti-miR-200c exerted opposite effects.

In an attempt to identify a miR-200c target involved in this process, we focused on FSTL1 for different reasons. FSTL1 has been demonstrated to be (1) a protective cardiokine, (2) involved in the EMT, (3) with multiple miRNA binding sites.

Wei and colleagues showed in mouse and swine MI models that epicardial FSTL1 declines following injury and its restoration through local injection of human FSTL1 by a bioengineered epicardial patch resulted in improved cardiac function, increased vessel formation, and increased cardiomyocyte proliferation [21].

These data suggest that an intrinsic epicardial FSTL1 suppressor may exist in the ischemic myocardium.

Therefore, we hypothesized that epicardial FSTL1 could be a target of miR-200c. This hypothesis was also supported by previous results demonstrating the presence of multiple miRNA-binding sites in the 3′UTR of FSTL1 mRNA and their role in the inhibition of FSTL1 expression [31,32,33,34]. Notably, it has recently been demonstrated that miR-9-5p is an intrinsic FSTL1 suppressor, and its neutralization stabilized FSTL1 expression and attenuated post-MI remodeling by restraining cell death and oxidation [34]. Comparing the expression levels of FSTL1 and miR-9-5p after MI, the authors also suggested that, except for this miRNA, other FSTL1-targeting molecules might exist in the heart.

Further, previous results already demonstrated that FSTL1 induced EMT in the airways of patients and mice by activating autophagy, and miR-524-5p/FSTL1 has the ability of regulating the progression of EMT of breast cancer cells [28,29]. Similarly, in our study, using an in vivo model of myocardial infarction in mice, we detected a strong upregulation of FSTL1 in epicardial cells undergoing EMT during acute MI. In addition, treatment with TGF β1 induced in EMCs in vitro a significant enhancement in the mRNA and protein expression levels of FSTL1.

Finally, a recent study showed that, under miR-137 regulation, FSTL1 maintained stemness in breast cancer cells via integrin b3/Wnt signaling. Accordingly, FSTL1 overexpression in these cells determined a significant enhancement of stem cell biomarkers as Sox2, Nanog, and CD133 [36].

To test our hypothesis, we firstly demonstrated by luciferase assay that FSTL1 is a direct target of miR-200c. Then, we showed that, in our in vitro EMT model, miR-200c overexpression in EMCs determines a significant inhibition of FSTL1 protein expression. Conversely, anti-miR-200c treatment was able to induce an increase of FSTL1 protein levels in EMCs. Therefore, our results validated miR-200c as a FSLT1-targeting miRNA that negatively inhibits its local expression.

It is noteworthy that we detected a significant modulation only of the non-glycosylated FSTL1 that has been demonstrated to be the form responsible, as secreted protein, for marked cardiomyocyte proliferation and cardiac regeneration in a mouse MI model [21,37]. In particular, Wei et al., studying the role of FSTL1 in cardiac regeneration, showed different effects on cardiomyocyte proliferation and protection from apoptosis due to the post-translational modification of the protein that depends on the type of cells in which FSTL1 is expressed: the epicardial non-glycosylated FSTL1 increased cardiomyocyte proliferation, while the myocardial hyperglycosylated protein, in accordance with previous reports [20,22], protected cardiomyocytes from apoptosis [21]. A more recent report showed that treatment with a modRNA construct encoding a mutant form of FSTL1 characterized by ablation of a single N-glycosylation site was sufficient and necessary to increase the proliferation of rat neonatal and mouse adult cardiomyocytes *in vitro* and after MI *in vivo*, respectively [37].

Our hypothesis was further strengthened by results obtained after FSTL1 silencing in EMCs. In particular, FSTL1 silencing demonstrated the involvement of this protein in EMC EMT, acquisition of EMT traits and enhancement in stem cell markers.

In conclusion, we hypothesized that not only secreted epicardial FSTL1, but also intrinsic epicardial FSTL1, might play an important role by inducing EMT and formation of cardiac progenitor cells that, *in vivo*, directly or through the secretion of paracrine factors might favor cardiac repair and regeneration.

Further, we propose that miR-200c represents an intrinsic FSTL1 suppressor, and its silencing may further enhance epicardial EMT, also suggesting a potential regenerative capability following MI.

## 4. Materials and Methods

### 4.1. Animal Model and In Vivo Study

MI was induced in C57BL/6J mice as previously described [38]. Briefly, in mice under anesthesia (100 mg/kg ketamine and 1 mg/kg acepromazine) and mechanically ventilated, thoracotomy via the third left intercostal space was performed. Then, the left coronary artery was ligated. The chest was closed and mice were allowed to recover. Sham operated mice were treated similarly, except that the ligature around the coronary artery was not tied. Animals were sacrificed 3 days after surgery. The remote, the border, and infarcted regions of infarcted or the left ventricle (LV) of sham operated hearts were collected and stored for WB analysis or in TRIzol reagent (Invitrogen, Carlsbad, CA, USA) to be processed for total RNA extraction; specifically, the infarcted area was recognized as a pale zone caused by gross necrosis of the myocardium, due to interruption of the blood supply to the area, while the border zone was identified as the area between the end of necrosis and the septum and the remote zone as the myocardial tissue opposite to the infarct zone.

### 4.2. Epicardial Cell Isolation

Epicardial cells were isolated from sham operated and 3-day infarcted hearts of 2–to 3-month-old C57Bl/6J female mice. Hearts were removed aseptically and, after apex resection, washed with ice-cold PBS. After rinsing, the hearts were placed in 0.25% trypsin–EDTA (0.25 trypsin, 1 mM EDTA in PBS) at room temperature while shaking at ~60 rpm. After 20 min, the epicardial surface of the heart was gently scraped, and the resulting cells were suspended in PBS plus 10% heat-inactivated fetal calf serum (FCS) to inactivate trypsin. After epicardial scraping, epicardial cells, as well as epicardium-depleted hearts of sham and infarcted hearts, were collected and stored at –80 °C in TRIzol reagent (Invitrogen, Carlsbad, CA, USA) to be processed for total RNA extraction.

### 4.3. Cell Culture and Treatment Protocols

A stable cell culture of mice Epicardial Mesothelial Cells (EMCs) was obtained, adapting the protocol by Eid and colleagues [39]. Specifically, epicardial cells, obtained after scraping (see “Epicardial Cell Isolation” for details) the epicardial surface of 2-to 3-month-old C57Bl/6J female mice, were centrifuged at 350 g for 6 min, and then resuspended in Dulbecco’s Modified Eagle’s Medium with high glucose (DMEM) (Sigma-Aldrich, St. Louis, MO, USA), 2 mmol/L l-glutamine (Sigma-Aldrich, St. Louis, MO, USA), 20% heat-inactivated fetal calf serum (FCS) (Sigma-Aldrich, St. Louis, MO, USA), and 100 units/mL penicillin and 0.1 mg/mL streptomycin (Sigma-Aldrich, St. Louis, MO, USA), and plated. The medium was changed after 1 h to remove non-attached cells and subsequently every 3–4 days. To promote their growth in culture, EMCs were plated on top of 3T3 irradiated (60 Gy for 1.5 min) fibroblasts (NIH/3T3, Cell Line murine, ECACC, Sigma-Aldrich), allowed to proliferate, and separated from the feeder layer using 0.05% trypsin before passaging [39]. Once separated from fibroblasts, EMCs were maintained in DMEM 10% fetal bovine serum (FBS) (Sigma-Aldrich, St. Louis, MO, USA) and maintained at 37 °C in a humidified atmosphere of 5% CO_2_ and 95% air. Cells were passaged at 80–90% confluence with 0.25% trypsin-EDTA and used between passages 16–30.

#### 4.3.1. TGF β1 Treatment

EMCs were plated (2 × 10^5^ cells) in 100 mm dishes in complete culture medium. After a 24 h starvation, cells were added with TGF β1 (Recombinant Mouse TGF β1, R&D Systems, Minneapolis, MN, USA) freshly prepared in complete medium at a final concentration of 0.5 ng/mL, 1 ng/mL, or 10 ng/mL, and incubated for 48 h, 72 h, or 96 h. Either total RNA or proteins were extracted from the cells.

#### 4.3.2. Stable Modulation of miR200c Expression

Stable overexpression of miR-200c-3p in EMCs was generated, after subcloning miR-200c-3p sequence in a proper lentiviral vector, by viral infection using lentiviral supernatants. miR-scramble was subcloned into the same lentiviral vector, as control. These viruses were produced as previously described [40]. In summary, EMCs were infected with lentiviral virus for 2 h and then were recovered in complete fresh medium for 24 h. Afterward, infected cells were selected by puromycin-containing medium (4 ug/mL of puromycin) for at least 72 h. miR-200c-3p overexpression was confirmed by qRT-PCR. miR-200c-3p overespressing and miR-scramble EMCs were then treated with 1 ng/mL TGF β1 for 24 h, before being collected for protein extraction and analysis. To obtain a stable downmodulation of miR-200c-3p expression, locked nucleic acid (LNA) oligonucleotides probes for miR-200c-3p or negative control oligonucleotides (miRCURY LNA MicroRNA detection probes; Exiqon, Vedbaek, Denmark) were transfected by siRNA transfection reagent (Santa Cruz Biotechnology, Dallas, TX, USA) in 40% confluent EMCs (5000 cells/cm^2^) at the final concentration of 40 nM. The expression level of miR-200c-3p was evaluated by qRT-PCR in EMCs transfected with anti-miR-200c-3p and compared with control sequence transfected cell; cells were then collected for protein extraction and analysis.

#### 4.3.3. Generation of FLST1-Silenced EMCs

To achieve FSTL1 transient knockdown, pre-designed siRNA targeting the mouse FSTL1 gene was purchased from Gene Solution siRNA from Qiagen (SI 01006222; Qiagen, MD, USA). Sequence of the siRNA oligonucleotide targeting the coding region of the mouse FSTL1 mRNA was AAGGTGCTATTTCTCTGTAAA. The negative control siRNA contained a 19-bp scrambled sequence with 3′dT overhangs (Qiagen, MD, USA). EMCs were transfected with siRNA by Hi Perfect Transfection Reagent (Qiagen, MD, USA) according to the manufacturer’s protocol. Cells were seeded in 24-well plate (4 × 10^4^ cells/well) in 400 µL of complete medium and immediately transfected for 48 h or 72 h with siFSTL1 or siControl. Transfection mixtures containing 3 µL/well of Hi PerFect Transfection Reagent, 100 µL/well of DMEM deprived of FBS, and a final concentration of 10 nM FSTL1 siRNA or siControl were incubated at room temperature for 10 min and then added to EMCs seeded before. When both siRNA (or siControl) and TGF β1 were used together, 1 ng/mL TGF β1 was added the day following siRNA transfection. Cells were harvested 24 h and 48 h after TGF β1 treatment, before being collected for protein extraction and analysis or before being used for the scratch assay.

### 4.4. In Vitro Cell Proliferation Assay

The effect of TGF β1 treatment on cell proliferation was tested by Trypan Blue exclusion assay. Briefly, 4–5 × 10^4^ cells/well were seeded in triplicate in a 12-well plate. After TGF β1 treatment (0.5 ng/mL, 1 ng/mL, or 10 ng/mL TGF β1 for 48 h, 72 h, and 96 h), cells were washed in PBS, detached with Trypsin-EDTA, and the living cell number was determined using Trypan Blue exclusion assay counting 10 field/well in a Neubauer Improved Chamber.

### 4.5. Quantitative Real-Time Polymerase Chain Reaction (qRT-PCR)

#### 4.5.1. Total RNA Isolation and Quantification

Total RNA was extracted with TRIzol reagent (Invitrogen, Carlsbad, CA, USA) according to the manufacturer’s recommendations from the following samples: (1) EMCs treated with TGF β1; (2) epicardial cells (*n* = 3/group, each *n* = pool of 6 animals) or epicardium depleted hearts (*n* = 3/group) from sham and infarcted hearts three days after coronary artery ligation as described [41]; (3) the left ventricle or the border zone, the remote zone and infarcted region, respectively, of sham and three day infarcted hearts (*n* = 3/group). Quality of RNA was checked using the Agilent 2100 Bioanalyzer and Nanodrop 1000.

#### 4.5.2. Quantitative Gene Expression Analysis

Approximately 750 ng to 1.5 μg of RNA was used in first strand cDNA synthesis using random primers and Superscript III First-Strand Synthesis Super Mix for qRT-PCR (Invitrogen, Carlsbad, CA, USA). A 20 μL reaction system, which included the specific primers, cDNA template (7.5 ng/reaction), and SYBR Green PCR mixture (Applied Biosystems, Foster City, CA, USA), was prepared for amplification of the cDNA of the genes of interest. The qRT-PCR reaction conditions were as follows: initial denaturation at 95 °C for 15 s, followed by annealing/extension at 60 °C for 1 min, for a total of 40 cycles. The amplification process was carried out on the 7900HT Real-time PCR system (Applied Biosystems, Foster City, CA, USA). All reactions were carried out in triplicate. The threshold cycle (CT), which correlates inversely with the levels of target mRNA, was measured as the number of cycles at which the reporter fluorescence emission exceeds the preset threshold level. The amplified transcripts were quantified using the comparative CT method with the formula for relative fold change = 2^−ΔΔCT^. GAPDH expression was used as internal control and for normalization. The mean was calculated, and possible significant differences were analyzed using Student’s *t* test. *p* < 0.05 was considered significant.

The primers used are reported in Table 1.

#### 4.5.3. Quantitative Mature miRNA Expression Analysis

qRT-PCR analysis of miRNAs was performed using the 7900HT Real-Time PCR System (Applied Biosystems, Foster City, CA, USA) and the miScript PCR System (Qiagen, ML, USA) according to the manufacturer’s instructions. Approximately 2 µg of total RNA was used in first strand cDNA synthesis (Qiagen, MD, USA). The resulting cDNA was then diluted to have 0.5 ng/μL input material for miRNA detection. A 20 μL reaction system, which included the specific primers (Qiagen, MD, USA), cDNA template (3 ng/reaction), and SYBR Green PCR mixture (Qiagen, MD, USA), was prepared for amplification of the cDNA of the miRNA of interest. Moreover, miRNA reverse transcription control (miRTC) (Qiagen, MD, USA) was performed to assess the performance of the reverse transcription reaction. The qRT-PCR reaction conditions were as follows: the reactions were initially incubated at 95 °C for 15 min, followed by 40 cycles of 94 °C for 15 s (denaturation), 55 °C for 30 s (annealing), and 70 °C for 30 s (extension). The amplification process was carried out on the 7900HT Real-time PCR system (Applied Biosystems, Foster City, CA, USA). All reactions were carried out in triplicate. The threshold cycle (CT), which correlates inversely with the levels of target mRNA, was measured as the number of cycles at which the reporter fluorescence emission exceeds the preset threshold level. The amplified transcripts were quantified using the comparative CT method with the formula for relative fold change = 2^−ΔΔCT^. snoRNA202 expression was used as an endogenous control and for normalization. The mean was calculated, and possible significant differences were analyzed using Student’s *t* test. *p* < 0.05 was considered significant.

### 4.6. Western Blot Assay

Total proteins from EMCs and LV from control mice were homogenized and extracted using RIPA buffer (10 mM Tris-HCl pH 7.4, 150 mM NaCl, 1% NP40, 1% Deoxycolic acid, 0.1% SDS and 10% Glycerol) containing protease and phosphatase inhibitors (2 mM phenylmethylsulfonyl fluoride, 100 U/mL of aprotinin, 10 μg/mL of leupeptin and pepstatin, 10 mM sodium fluoride, 20 mM sodium vanadate). Equal amounts of total cellular proteins (50 μg/lane) were resolved by SDS-polyacrylamide gel electrophoresis and transferred to nitrocellulose membrane (Amersham Pharmacia Biotech, Little Chalfont, UK). 

Membranes were probed with rabbit monoclonal anti-total Smad 2/3 (#8685, Cell Signalling Technology, Danvers, MA, USA), rabbit monoclonal anti-Phospho Smad 2 (Ser 465/467) (#3108, Cell Signalling Technology), rabbit monoclonal anti-Phospho Smad 3 (Ser 423/425) (#9520, Cell Signalling Technology), goat polyclonal anti-Tbx18 (sc-17869, Santa Cruz Biotechnology, Santa Cruz, CA, USA), mouse monoclonal anti-Cardiac Troponin T (sc-20025, Santa Cruz Biotechnology), mouse monoclonal anti-PECAM1 (CD31) (sc-376764, Santa Cruz Biotechnology), mouse monoclonal anti-GAPDH (sc-137179, Santa Cruz Biotechnology), rabbit polyclonal anti-Follistatin-like 1 (FSTL1) (MBS713792, MY BioSource, San Diego, CA, USA), rabbit monoclonal anti-TCF8/ZEB (D80D3) (3396S, Cell Signaling Technology), mouse monoclonal anti-VCAM1 (sc-13160, Santa Cruz Biotechnology), mouse monoclonal anti-Vimentin (sc-373717, Santa Cruz Biotechnology), mouse monoclonal anti-Oct4 (sc-5279, Santa Cruz Biotechnology), mouse monoclonal anti-Nanog (sc-374103, Santa Cruz Biotechnology), mouse monoclonal anti-Calponin 1 (sc-58707, Santa Cruz Biotechnology), mouse monoclonal anti c-kit (sc-365504, Santa Cruz Biotechnology), mouse monoclonal anti-Sox2 (sc-365823, Santa Cruz Biotechnology), mouse monoclonal anti-α smooth-muscle-actin (αSMA) (Ab-7817, Abcam, Cambridge, UK), and rabbit polyclonal anti-CDK4 (C-22) (sc-260, Santa Cruz Biotechnology), followed by horseradish peroxidase-coupled secondary antibodies and developed by a chemiluminescence-based detection system (ECL, LiteAblot Turbo, EuroClone). 

### 4.7. Immunofluorescence

For immunofluorescence staining, EMCs (4 × 10^4^) were seeded onto coverslips placed on the bottom of a 24-well plate and incubate as follows: with DMEM 10% FBS for 48 h for EMCs characterization; with or without 1 ng/mL TGF β1 for 48 h for Filamentous actin (F-actin) (Phalloidin staining) and stem cell-like marker evaluation; with siFSTL1 or siControl for 72 h together with TGF β1 (or not) for 48 h, as previously described, for EMT analysis. Cells were then fixed for 15 min with 4% paraformaldehyde in PBS, washed twice in PBS and permeabilized for 10 min with 0.5% Triton X-100 in PBS. After 1 h block with 1% bovine serum albumin (BSA) at room temperature, coverslips were incubated in a humidified chamber at 4 °C overnight with the following primary antibodies: mouse monoclonal anti-β catenin (sc-7963, Santa Cruz Biotechnology), mouse monoclonal anti-WT1 (sc-7385, Santa Cruz Biotechnology), mouse monoclonal anti-α smooth-muscle-actin (αSMA) (Ab-7817, Abcam) and rabbit polyclonal anti-ZO-1 (40-2200, Invitrogen), mouse monoclonal anti-Oct4 (sc-5279, Santa Cruz Biotechnology), mouse monoclonal anti-Nanog (sc-374103, Santa Cruz Biotechnology) and rabbit monoclonal anti c-kit (ab231780, Abcam).

Afterwards, coverslips were washed with PBS for 3 times (5 min/wash), and incubated for 1 h, respectively, with a goat anti-mouse IgG Alexa Fluor 555 and a goat anti-rabbit IgG Alexa Fluor 555 fluorescent secondary antibodies (1:200, Invitrogen, Carlsbad, CA, USA).

Filamentous actin (F-actin) was stained with phalloidin-TRITC (P1951, Sigma-Aldrich) diluted 1:200 for 20 min after 1% BSA blocking for 1 h.

Finally, samples were washed with PBS 3 times (5 min/wash), and nuclei were stained using SYTO green fluorescent nucleic acid staining (1:10,000, 5 min; Molecular Probes, Eugene, OR, USA). Coverslips were mounted in ProLong Diamond Antifade Mountant (Life Technologies, Thermo Fisher Scientific, Carlsbad, CA, USA). For the analysis of the immunofluorescence, cells were imaged on LSM 501 confocal microscopy equipped with a digital camera (Zeiss, Oberkochen, Germany).

### 4.8. Luciferase Assay

HEK 293 cells were plated in 12-well plates and were transfected with either 0.25 μg of the full-length 3′-UTR luciferase construct pEZX-MT06-3′UTR-FSLT1 (NM_008047.5) or the mutated one pEZX-MT06-3′UTR-FSLT1 mut, in which the seed sequence AGTATT spanning from position 93–99 nt of the 3′UTR was mutated in CCGCTT. The constructs were co-transfected with either 0.25 μg of plKO.1-pre-miR-200c or plKO.1-scramble. Cellular extracts were tested with Dual Luciferase Assay (Promega, Madison, WI, USA) according to the manufacturer instructions, using an EnSight plate reader (Perkin Elmer, Waltham, MA, USA). Values were normalized according to renilla luciferase activity. 

### 4.9. Scratch Assay

EMCs were seeded into 12-well plates at a density of 6 × 10^4^ cells per well and treated with siRNA and TGF β1 as previously described. After cells reached 100% confluency, the adherent cell layer was wounded by scraping two crossing perpendicular lines with a sterile 10 µL tip. Then, after a rapid wash with PBS to eliminate detached cells, fresh low-serum (2%) DMEM was added to cells. Wounds were observed and photographed under a Zeiss IM35 microscope (Zeiss) equipped with a digital camera (Nikon Digital Sight DS-L1) at 0, 12, 24, 32, and 48 h after the scratch was made. The distance between the leading edges of the migrating EMCs was measured using ImageJ software (National Institutes of Health, Bethesda) and normalized to 0 h at four sites in each image.

Cell migration was determined using the following formula:

$$\mathrm{Percentage}\;\mathrm{of}\;\mathrm{wound}\;\mathrm{healing}\;\left(\%\right)=[\frac{100-\left(\mathrm{wound}\;\mathrm{width}\;\mathrm{at}\;\mathrm{the}\;\mathrm{observed}\;\mathrm{time}\;\mathrm{point}\right)}{\mathrm{wound}\;\mathrm{width}\;\mathrm{at}\;\mathrm{the}\;0\;h}]\times 100$$

Statistical analysis was performed using unpaired Student’s *t*-test.

### 4.10. Data Collections and Statistics

Parametric statistical analysis was performed using Student’s t-test for two groups; two-way analysis of variance (ANOVA) was applied for multiple comparisons with Bonferroni post hoc analysis. Differences between groups were considered statistically significant at values of *p* < 0.05. Results are expressed as mean ± S.E.M. Data were analyzed using GraphPad version 9 for Windows (La Jolla, CA, USA). 

## Figures and Tables

**Table 1:** List of primers used in this study.

Gene	Forward Primer	Reverse Primer
WT1	cagatgaacctaggagctaccttaaa	tgcccttctgtccatttca
TBX18	caccgaggccgacgaagacc	cgtcctcacagctgcccgc
TCF21	cattcacccagtcaacctga	ccacttccttcaggtcattctc
VCAM1	tcttacctgtgcgctgtgac	actggatcttcagggaatgagt
SNAI1	cttgtgtctgcacgacctgt	caggagaatggcttctcacc
SNAI2	cattgccttgtgtctgcaag	agaaaggcttttccccagtg
ACTA2	ctctcttccagccatctttcat	tataggtggtttcgtggatgc
SM22-alpha	ccttccagtccacaaacgac	gtaggatggacccttgttgg
VIMENTIN	tgcgccagcagtatgaaa	tgcgccagcagtatgaaa
FIBRONECTIN1	cggagagagtgcccctacta	cgatattggtgaatcgcaga
CALPONIN 1	cggcttgtctgctgaagtaa	accccctcaatccactctct
CYCLIN D1	agaaggagattgtgccatcc	ctcttcgcacttctgctcct
CKIT	gatctgctctgcgtcctgtt	cttgcagatggctgagacg
NANOG	gcctccagcagatgcaag	ggttttgaaaccaggtcttaacc
OCT4	gttggagaaggtggaaccaa	ctccttctgcagggctttc
SOX2	acggcagctacagcatga	gacgtcgtagcggtgcat
FLST1	cagccatcaacatcaccact	atgagggcgtcaacacaga
ZEB 1	aggtgatccagccaaacg	ggtggcgtggagtcagag
ZEB 2	ccagaggaaacaaggatttcag	aggcctgacatgtagtcttgtg
GAPDH	tgccaagtatgatgacatcaagaag	ggtcctcagtgtagcccaagat

## Data Availability

The datasets generated during and/or analyzed during the current study are available from the corresponding author on reasonable request.

## Supplementary Materials

The following are available online at https://www.mdpi.com/article/10.3390/ijms22094971/s1, **Figure S1:** Immunofluorescence of isolated and expanded EMCs in culture for the expression of polarized epithelial markers (A) β-catenin and (B) ZO-1. Left panel: red fluorescence indicates β-catenin or ZO-1. Central panel: green fluorescence indicates SYTO Green Fluorescent staining of nuclei. Right panel: merge of both images. Scale Bar: 50 µm. Expression of epicardial markers (C) WT1, (D) Tbx18, (E) Tcf21 and epithelial marker (F) VCAM1 in cultured EMCs. Relative qRT-PCR data were depicted relative to epicardial cells isolated from hearts (Epi MI 3d), 3 days following MI. Data were normalized to GADPH and represent means ± SEM (*n* = 3, each *n* = pool of 6 animals for Epi MI; *, *p* < 0.05 vs. Epi MI 3d). Immunofluorescence for WT1 (G). Left panel: red fluorescence indicates WT1. Central panel: green fluorescence indicates SYTO Green Fluorescent staining of nuclei. Right panel: merge of both images. Scale Bar: 25 µm. (H) Validation of EMC culture purity was performed by WB analysis, excluding contamination from myocytes, smooth muscle cells and endothelial cells. The EMC culture displayed positivity for the epicardial marker Tbx18 but neither for Troponin T nor for CD31 and showed only a marginal positivity for αSMA; **Figure S2**: (A) Phase-contrast microscopy of EMC cultures stimulated with TGF β1 (1 ng/mL) and examined after 48 h. The phosphorylation levels of Smad2 and Smad3 were detected by Western blot analysis. Western blot analysis showing the expression of (B) p-SMAD2 and p-SMAD3 in EMCs stimulated with 0.5, 1 and 10 ng/mL of TGF β1 for 48, 72 and 96 h compared to control conditions (CTRL). The same filter was probed with anti-CDK4 pAb to show the equal loading. Upper panel: A representative Western blotting of three independent experiments is shown. Lower panel: Densitometric analysis of Western blot. Data are shown as means ± SEM. *, *p* < 0.05 for 0.5 ng/mL of TGF β1; #, *p* < 0.05 for 1 ng/mL of TGF β1; §, *p* < 0.05 for 10 ng/mL of TGF β1 vs. control conditions (CTRL). **Figure S3**: Expression of miR-200c direct targets in EMCs. (A,B) mRNA expression of ZEB1 and ZEB2 (miR-200c direct targets) by real time PCR and (C) protein expression levels of ZEB1 by WB determined in EMCs exposed to 48, 72 and 96h of treatment with TGF β1 at three different concentrations (0.5, 1 and 10 ng) compared to control conditions (CTRL).*, *p* < 0.05 for 0.5 ng/mL of TGF β1; #, *p* < 0.05 for 1 ng/mL of TGF β1; §, *p* < 0.05 for 10 ng/mL of TGF β1 vs. control conditions (CTRL).
