# Supplementary figures and images for: miR-200c-3p Regulates Epitelial-to-Mesenchymal Transition in Epicardial Mesothelial Cells by Targeting Epicardial Follistatin-Related Protein 1
Source: Int J Mol Sci. 2021 May 7;22(9):4971. doi: 10.3390/ijms22094971 (PMC8125323; doi:10.3390/ijms22094971)

Figure S1

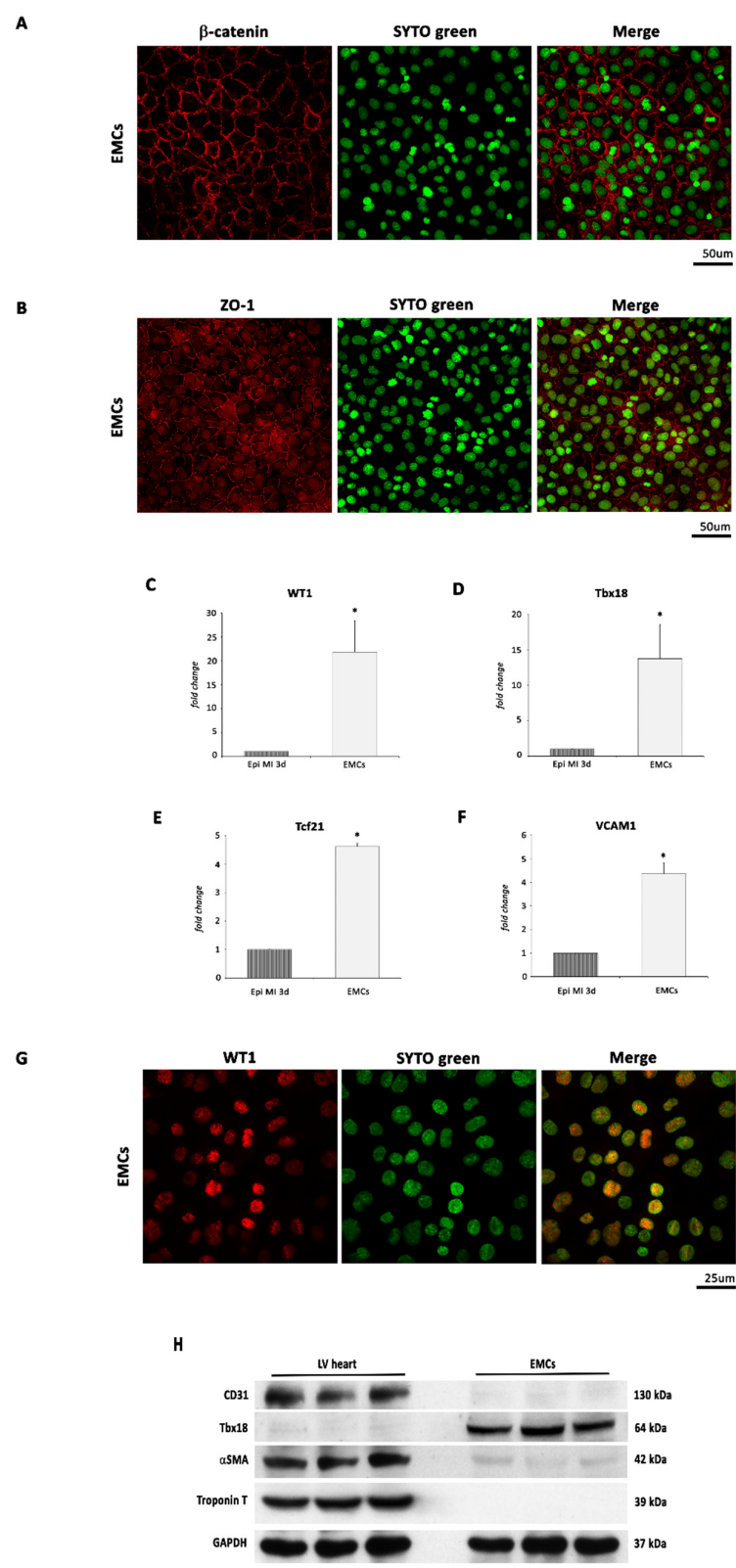

Figure S2

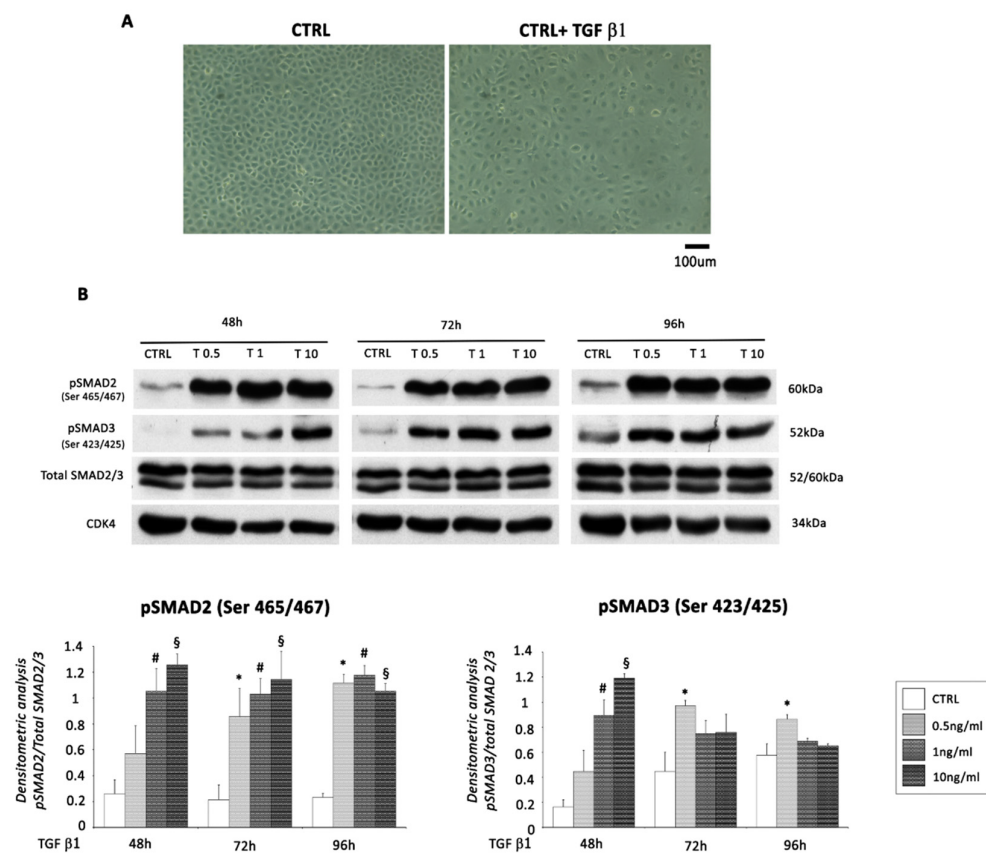

Figure S3

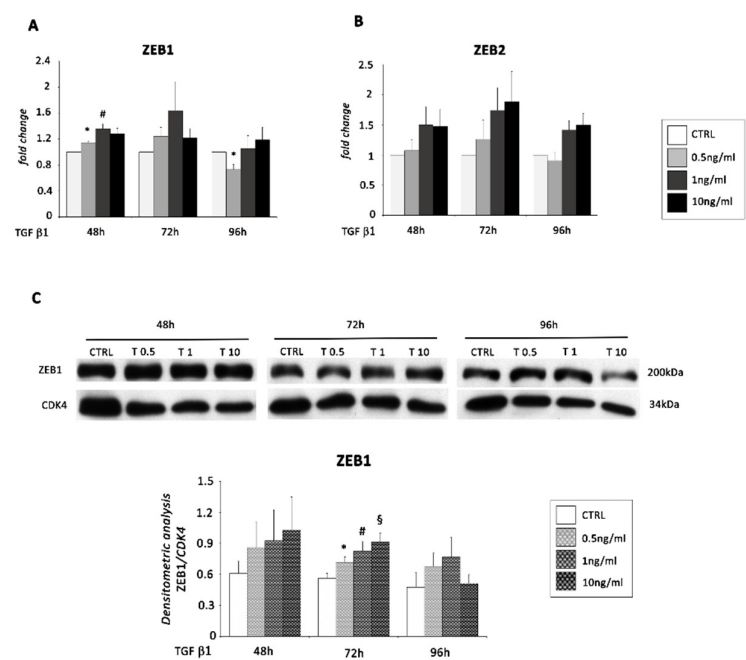

Supplement: Supplementary file 1 [file ijms-22-04971-s001.zip › ijms-1208621-supplemebntary Figure.pdf]
